# Patient Perspectives on Using a Smartphone App to Support Home-Based Exercise During Physical Therapy Treatment: Qualitative Study

**DOI:** 10.2196/35316

**Published:** 2022-09-13

**Authors:** Remco Arensman, Corelien Kloek, Martijn Pisters, Tjarco Koppenaal, Raymond Ostelo, Cindy Veenhof

**Affiliations:** 1 Center for Physical Therapy Research and Innovation in Primary Care Julius Health Care Centers De Meern Netherlands; 2 Physical Therapy Research, Department of Rehabilitation, Physiotherapy Science, and Sport Brain Center, University Medical Center Utrecht Utrecht University Utrecht Netherlands; 3 Expertise Center Healthy Urban Living Research Group Innovation of Human Movement Care HU University of Applied Sciences Utrecht Netherlands; 4 Research Group Empowering Healthy Behaviour Department of Health Innovations and Technology Fontys University of Applied Sciences Eindhoven Netherlands; 5 Department of Health Sciences, Faculty of Science VU University Amsterdam Amsterdam Movement Sciences Research Institute Amsterdam Netherlands; 6 Department of Epidemiology and Data Science Amsterdam University Medical Centre Amsterdam Netherlands

**Keywords:** patient perspectives, mobile health, mHealth, home-based exercise, adherence, low back pain, physical therapy

## Abstract

**Background:**

Home-based exercise is an important part of physical therapy treatment for patients with low back pain. However, treatment effectiveness depends heavily on patient adherence to home-based exercise recommendations. Smartphone apps designed to support home-based exercise have the potential to support adherence to exercise recommendations and possibly improve treatment effects. A better understanding of patient perspectives regarding the use of smartphone apps to support home-based exercise during physical therapy treatment can assist physical therapists with optimal use and implementation of these apps in clinical practice.

**Objective:**

The aim of this study was to investigate patient perspectives on the acceptability, satisfaction, and performance of a smartphone app to support home-based exercise following recommendations from a physical therapist.

**Methods:**

Using an interpretivist phenomenology approach, 9 patients (4 males and 5 females; aged 20-71 years) with nonspecific low back pain recruited from 2 primary care physical therapy practices were interviewed within 2 weeks after treatment ended. An interview guide was used for the interviews to ensure that different aspects of the patients’ perspectives were discussed. The Physitrack smartphone app was used to support home-based exercise as part of treatment for all patients. Data were analyzed using the “Framework Method” to assist with interpretation of the data.

**Results:**

Data analysis revealed 11 categories distributed among the 3 themes “acceptability,” “satisfaction,” and “performance.” Patients were willing to accept the app as part of treatment when it was easy to use, when it benefited the patient, and when the physical therapist instructed the patient in its use. Satisfaction with the app was determined by users’ perceived support from the app when exercising at home and the perceived increase in adherence. The video and text instructions, reminder functions, and self-monitor functions were considered the most important aspects for performance during treatment. The patients did not view the Physitrack app as a replacement for the physical therapist and relied on their therapist for instructions and support when needed.

**Conclusions:**

Patients who use an app to support home-based exercise as part of treatment are accepting of the app when it is easy to use, when it benefits the patient, and when the therapist instructs the patient in its use. Physical therapists using an app to support home-based exercise can use the findings from this study to effectively support their patients when exercising at home during treatment.

## Introduction

The effectiveness of exercise therapy in the treatment of musculoskeletal disorders has been studied extensively, and exercise therapy remains an important part of treatment in clinical practice [1]. However, treatment is not limited to supervised exercise. Home-based exercise (HBE) programs allow patients to exercise at home between visits to the clinic. Unfortunately, the effectiveness of HBE relies heavily on patient adherence, which has been shown to be low [2-5].

Different factors contribute to patient adherence to HBE, including several factors that can be easily influenced by a physical therapist [6,7]. For example, a physical therapist can not only provide support and positive feedback, but also follow up on exercise recommendations during future visits to reinforce patient adherence. Additionally, practitioners can increase patient adherence to HBE by recommending a feasible maximum of 2-4 exercises, supporting and improving self-efficacy, and supporting patients to incorporate exercise into their daily life [6]. These strategies aim to improve or reinforce patient adherence to the frequency, intensity, and quality of their performance of exercise recommendations. However, increasing adherence to HBE remains challenging even when employing different strategies.

Smartphone apps have the potential to provide new solutions to support adherence to exercise recommendations. Exercise apps using personalized exercise programs, video instructions, and reminders to exercise can increase adherence by providing performance guidance and remote support, and improving physical therapist–patient interactions regarding HBE [8,9]. Furthermore, apps supporting health behaviors provide health benefits and additional support in the patient’s own home environment [10,11]. Research has shown that patients with nonspecific low back pain (LBP) are mainly worried that despite the benefits of new technologies (eg, reminders and remote support), their use leads to less personalized care [12]. However, patients also expect these technologies to support HBE by increasing performance and adherence to exercise recommendations [12]. To our knowledge, and based on our review of the literature, no qualitative studies are available on patients who used an app to support HBE alongside physical therapy, highlighting an important gap in the literature.

With the increasing availability of apps to support physical therapy treatment, a better understanding of patient perspectives on using these apps during physical therapy can assist physical therapists to effectively tailor the use of these apps for their patients and consequently improve treatment efficacy. Therefore, the aim of this study was to investigate patient perspectives on the acceptability, satisfaction, and performance of an app to support HBE following recommendations from a physical therapist.

## Methods

### Design

This study was performed using qualitative methods associated with phenomenology and an interpretivist approach. Data were collected by interviewing a sample of patients with LBP who used Physitrack (Physitrack Limited) during treatment in a primary care physical therapy practice. 

### Ethics Approval

The Medical Research Ethics Committee of the University Medical Center Utrecht ruled that the Medical Research Involving Human Subjects Act does not apply to this study (protocol number 17-034/C). This study complies with the Declaration of Helsinki, and the standards for reporting qualitative research were followed in reporting this work [13].

### Study Procedures and Recruitment

**Figure 1:**
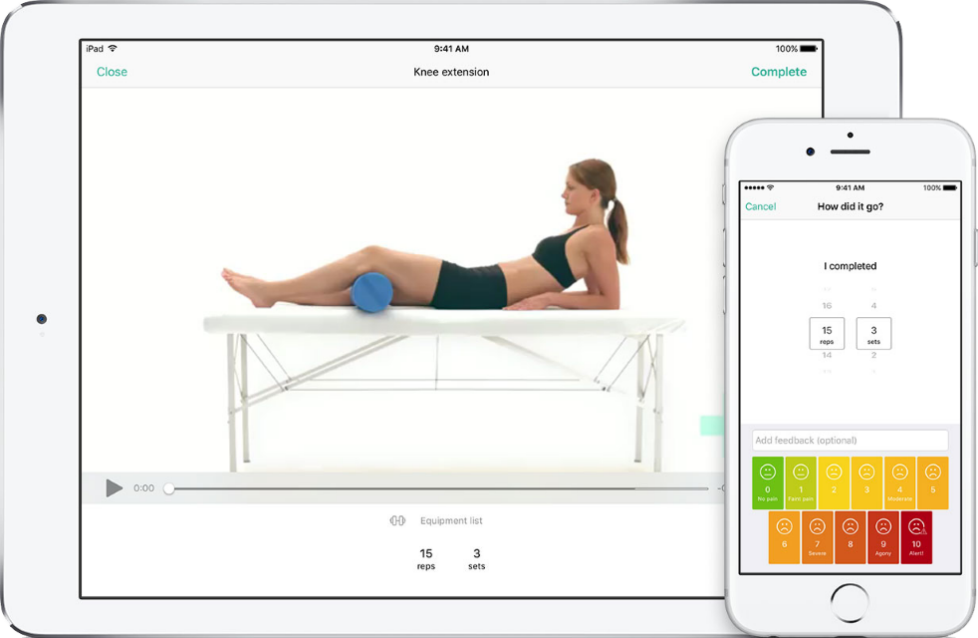
Examples of the Physitrack app used on a tablet and a smartphone.

**Figure 2:**
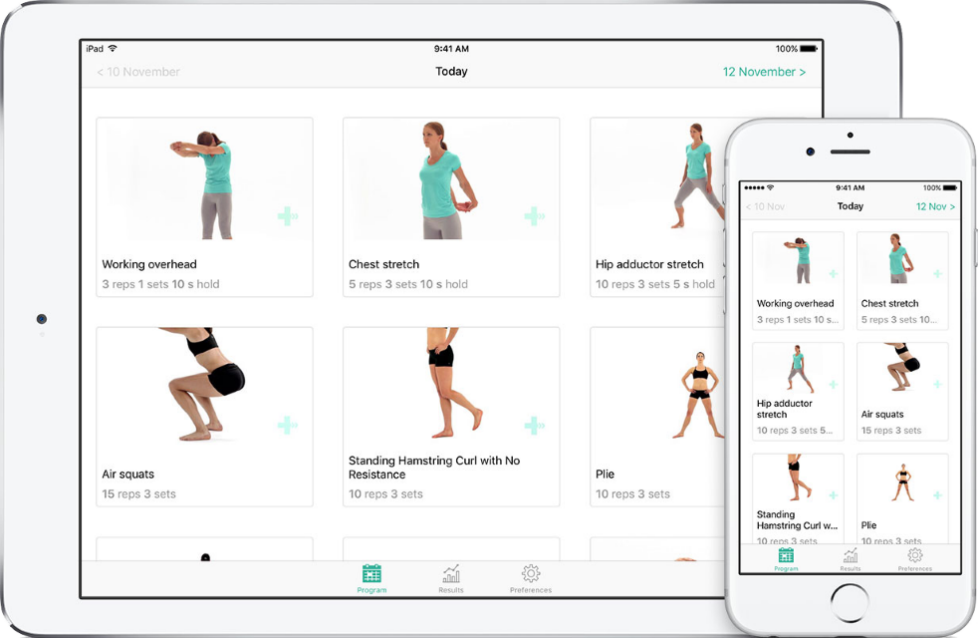
Examples of a home-based exercise program in the Physitrack app viewed on a tablet and a smartphone.

All patients were recruited from January to April 2018 from 2 participating primary care physical therapy practices in the Netherlands. For each participating practice, a physical therapist specializing in the treatment of spinal pain volunteered to recruit patients. Both physical therapists had 2 years of experience working with Physitrack. Physitrack allows physical therapists to create and share personalized exercise programs with patients through the Physitrack app, email, or paper handouts (see Figures 1 and 2 for examples). The app allows patients to set reminders to perform their exercises, track their adherence, rate pain scores during the exercises, and send direct messages to their physical therapists. To be eligible for participation, a patient had to have been treated by one of the participating physical therapists, their treatment had to have ended less than 2 weeks prior to participation in the study, and the physical therapist had to have sent the patient HBE recommendations using the Physitrack app during treatment. Patients were excluded if they had insufficient command of the Dutch language for casual conversation. Patients interested in the study were contacted by a researcher (RA) and were provided with information about the study and procedures. An appointment for the interview was made with interested patients, and written informed consent was obtained prior to the interview. A purposive sampling method was chosen to include a heterogeneous sample based on age and gender. Additionally, the participants were asked to complete the Systems Usability Scale (SUS) to provide an objective measure of usability for Physitrack [14]. The SUS consists of 10 items rated on a 5-point scale ranging from strongly agree to strongly disagree. The SUS score ranges from 0 to 100, and usability of the app is acceptable for ratings of 70 or higher [15]. The goal was to recruit similar numbers of males and females with a high variation in age until saturation of the data was achieved. Data saturation was reached when new data repeated previous data without adding new information, and saturation was checked during data analysis in an iterative process [16].

To guide the interviews, an interview guide based on the conceptual framework for testing electronic adherence monitoring devices was used [17]. The conceptual framework contains an objective dimension and a subjective dimension. Because the focus of this study was on patients’ perceptions, only the subjective dimension and the components performance, satisfaction, and acceptability were used [17]. A first draft of the interview guide was created and refined using feedback from an expert meeting consisting of 15 researchers from the Physiotherapy Science research group at Utrecht University. Additionally, 5 physical therapists from the Leidsche Rijn Julius Healthcare centers were consulted to further refine and improve the interview guide. All researchers and physical therapists involved in this stage had experience working with mobile health (mHealth) apps in clinical practice, developing mHealth apps for other patient groups (eg, patients after stroke, patients with osteoarthritis, and those with musculoskeletal complaints), or both.

### Interviewer

All interviews were performed by a trained research assistant with a background in physical therapy and prior experience conducting interviews. The interviewer received an additional 2-hour training in qualitative interviewing techniques, and 2 pilot interviews were performed, recorded, and discussed with a researcher (RA) to ensure the thoroughness of the interviews. During data collection, the interviewer discussed each completed interview with the same researcher to ensure consistency between interviews.

### Interviews

The interviews were conducted in a private room in the practice where the participant had received treatment. The research assistant audio recorded and transcribed each interview verbatim. A researcher (RA) checked the transcription for accuracy using the interview recording, after which a written summary of the interview was sent to the participant for a member check. The participant was asked to read the summary and provide additional information or corrections when the summary did not properly reflect their perspectives. None of the participants requested changes to their interview during the member check.

### Data Analysis

The transcripts were anonymized and subsequently analyzed using the “Framework Method” [18]. This approach consists of 7 stages, namely transcription, familiarization with the interviews, coding, development of a working analytical framework, application of the analytical framework, charting of data into the framework matrix, and interpretation of the data. The goal was to describe the common experiences and perspectives of the participants. Stages 1 and 2 were completed during data collection.

An “inductive coding” approach was chosen for stage 3, the coding stage, and Microsoft Excel 2016 was used to aid with the analysis. Coding was performed by extracting meaningful quotes from the transcripts to an Excel datasheet, adding a short descriptive code to the quote, grouping related or similar quotes, and repeating the process until the entire transcript was coded. The first 3 interviews were independently coded by 2 researchers (RA and CK) [19]. After an interview was coded, the researchers compared results and discussed differences in coding until they reached a consensus, and they labeled the codes with a short descriptive name. If the researchers could not reach a consensus, a third researcher (MP) was consulted. The remaining interviews were coded by 2 researchers (RA and CK) working together. During the coding process, the researchers continuously refined and adjusted the codes to best fit the data.

In stage 4, paper prints of the codes and their associated quotes from the first 3 interviews were used to allow a hands-on approach for the creation of categories and an initial analytical framework. Categories were formed by grouping codes that appeared to be related until all codes were assigned to a category. The categories were then grouped under themes based on the topics from the interview guide. To reduce bias introduced by the personal perspectives of a single researcher, the researchers (RA and CK) worked together to construct the framework and discussed each new category and its place within the framework until they reached a consensus. The analytical framework was continuously developed in an iterative process. Categories were merged, split, or relabeled, and codes were assigned to different categories in an attempt to best fit the data until all interviews were analyzed. After each iteration, the members of the research team (RA, CK, MP, TK, RO, and CV) discussed the new framework matrix and used the input from the discussion for the next iteration. The final framework matrix contained all categories with the summarized data from each interview and was used to interpret the data, completing stages 6 and 7 of the analysis.

## Results

### Participant Characteristics

Once data saturation was reached after 9 interviews, recruitment ended. The characteristics of the patients included in the study can be found in Table 1.

**Table 1:** Participant characteristics.

Participant number	Gender	Age (years)	SUS^a^ score (0-100)
1	Male	42	70
2	Female	29	82.5
3	Male	39	90
4	Female	33	90
5	Female	38	92.5
6	Female	45	97.5
7	Female	52	77.5
8	Male	71	85
9	Male	20	92.5

^a^SUS: System Usability Scale.

Data analysis revealed 11 categories distributed among the 3 themes “acceptability,” “satisfaction,” and “performance.” “Acceptability” describes what was required for participants to accept the app as part of their treatment. The categories grouped under “satisfaction” describe the perceived benefits of using the app during treatment. The theme “performance” contains a single category with the same name and describes the most important app functions according to the participants, as well as suggestions to improve the performance of the app.

### Acceptability

#### Usability

The app was easy to use, according to the participants. The app was simple in design, which made it very accessible.

I think it just has to be simple, without too many bells and whistles, and for me, it worked like that.

Participant #3

#### Availability

The availability of the exercises on the patients’ smartphones was perceived as an advantage because using a smartphone was already integrated into their daily lives. None of the participants experienced the requirement to own a smartphone in order to use Physitrack as a problem.

It’s just very easy. You carry your phone with you every day anyway, so when you forget something, you can just open the app and find it; very easy.

Participant #7

#### Willingness to Use the App

Participants were unaware that Physitrack existed before starting treatment, but all were willing to try the app to see if it would be useful for them. The perceived benefit from using the app during treatment determined its continued use for the participants.

I didn’t have any expectations, and I went pretty open-minded into it. I thought that if it adds anything, it’s great, but if it doesn’t, I can just remove it from my phone.

Participant #2

Although patients were open-minded, perceived privacy issues were a concern for participant #1.

After reinstalling the app on my phone, I had to look through my old e-mails to find the login code, and it’s, of course, strange that if anyone else gets his hands on that e-mail, they can see all my exercises and my private information.

Participant #1

#### Importance of Instructions

Participants found it essential to be taught how to use the app and told which functions of the app are important for them. The interviewees saw the physical therapist as the person responsible for properly instructing patients in the use of the app.

I only used the videos because the physical therapist showed me, but I didn’t look for any other options. I think that if you want to use all the functions of the app, the physical therapist has to explain them or provide a manual or something.

Participant #4

Patients rarely mentioned experiencing problems when using the app, suggesting that instructions by the physical therapist were sufficient to use the app in daily life. The only issues mentioned were setting the reminder for the exercises and not receiving the reminders.

After checking, I found that reminders were turned off, which is odd since I turned the reminders on and then didn’t get any.

Participant #1

### Satisfaction

#### Being Reminded

The reminder messages for the app’s exercises helped almost all participants to exercise more often or more regularly than they expected to without using the app.

In my busy life, the reminders motivated me to take some time to get it done.

Participant #4

Only participant #6 found the reminders useless, as they would come at inconvenient moments, even though the participant chose the time for the reminders.

Nine out of ten times when I set a reminder, I don’t get to doing it anyway, so I just turned them off after a while.

Participant #6

#### Feeling Supported

Being able to review the exercise recommendations at home and having something to fall back on were positive experiences and gave the patients the feeling that the app was supporting them.

After listening to the therapist, I would come home and still have questions or forgot what the therapist said. Then, I had something to fall back on, and that was very pleasant.

Participant #8

#### Satisfaction With Own Adherence

Participants were delighted with their adherence to the exercise recommendations and felt that the app helped them exercise as often as recommended and correct their performance.

The app helped with exercising. Not because I forgot them, … but I could check which exercises I had to do and how often.

Participant #5

Thanks to the app, I could see what exactly it was I was supposed to do … That definitely increased how often I exercised.

Participant #9

Although the app supported the patients with exercising, usage of the app generally declined quickly when exercises remained the same or when complaints were resolved.

The first time, I watched all the videos and memorized them. After that, I think I read the instructions for the exercises once or twice, but mostly used the app for the reminders.

Participant #5

I used the app only when new exercises were added because I already knew the others.

Participant #6

#### Supporting Treatment

Patients considered the use of the app to record problems, adherence, or pain scores or the use of the chat function to ask a quick question as contributing to the quality of the treatment. The physical therapist had access to information recorded by the patient between therapy sessions and could use it to personalize treatment for the patient. Participants saw the app as something to combine with the expertise of the physical therapist rather than a replacement. The physical therapist used the face-to-face treatments to adjust and personalize the HBE program, and the participants used the app to bring the support from their physical therapist into their own homes.

First, we practiced the exercises together, then I received the app, and the next week the therapist asked me how it went. If I had any problems, I could discuss them with him so he could change the exercise program for me.

Participant #7

The app is good progress, but it’s not yet a replacement of the physical therapist.

Participant #8

#### Quality of Exercise Performance

Patients felt that the app helped to improve their performance of the recommended exercises and perceived the app as a tool to maintain the quality of performance expected from them by the physical therapist. The visual examples of the app’s exercises appeared to increase self-efficacy and might have increased adherence.

There was one exercise I had trouble doing right, so if I didn’t have the video, I probably wouldn’t have remembered how to do it and probably wouldn’t have done it at all.

Participant #3

I wouldn’t say it improves how you do it if you already did it well. But it does make sure you don’t do it worse. It helps to keep the quality high.

Participant #9

#### Self-monitoring

Not all patients mentioned recording pain or adherence to exercises in Physitrack. However, patients who did record these metrics used the information to monitor their progress or demonstrate to the physical therapist that they had followed the exercise recommendations.

I felt that my back was very painful this week, but actually my pain score after doing the exercises is decreasing. That is, for me, a reminder I’m going in the right direction, and I find that very reassuring.

Participant #2

### Performance

According to the patients, the most appreciated or essential functions of Physitrack were the video and text instructions and the reminder function. Recording and monitoring their own progress and the chat function were mentioned less often but were still considered important by several patients.

Something that should stay in the app is this overview with all the videos and the names of the exercises and how often I’m supposed to do them. Together with the reminder, I think those are important.

Participant #5

The patients also suggested several improvements for the app, including connecting the app with the calendar on users’ mobile phones, such that follow-up visits could be automatically entered into the calendar. Other suggestions included repeated reminders when exercise performance was not recorded in the app, the option to connect the exercise videos to the television, and a loop or timer in the videos so that the patient could exercise along with the video.

## Discussion

### Principal Findings

The aim of this study was to investigate patient perspectives regarding an app to support HBE recommended by a primary care physical therapist. Qualitative data analysis revealed 11 categories describing the 3 themes of “acceptability,” “satisfaction,” and “performance.”

The “acceptability” theme contains the subthemes of usability, availability, willingness to use the app, and importance of instruction, and it describes what the patients perceived as essential to accept the app as part of treatment. Participants commented on how easy or difficult it was to use the app in their daily lives. Patients’ acceptance and continued use of the app as part of treatment appear to be based mainly on the perceived benefit. When a patient did not perceive or no longer saw any benefit from using the app, use declined quickly. The participants unanimously agreed that Physitrack was easily integrated into their daily routine. Although none of the participants had previously used Physitrack or a similar app during physical therapy, the app was accepted by all participants. Unfortunately, the quick and easy acceptance of a new mHealth app is not always reliable and depends on several different factors such as “perceived usefulness,” “social influence,” and “attitude” [20,21].

The acceptance of Physitrack in this study was possibly realized by the combination of the physical therapist introducing the app as part of treatment and the ease of use of the app. Even when a participant no longer found the app useful, it was very easy for them to stop using the app. As a result, there was no downside for the participant to try the app, as they could decide on its usefulness and continued use later on.

The participants felt that more instructions from their physical therapist were needed for optimal use of the app. The participants viewed the app as part of treatment and therefore relied on the physical therapist to provide guidance and support. Similarly, when participants experienced a problem using the app, they relied on the physical therapist for assistance. This finding underlines the importance of instructions, personal contact, and support from a physical therapist during treatment when using apps such as Physitrack [22]. It appears that part of the success of the integration of Physitrack into treatment relies on patient-therapist interaction. This is further supported by previous findings that the diagnosis of the patient does not seem to significantly impact the acceptance of mHealth apps during treatment [20].

“Satisfaction” describes the perceived benefit of using the app during treatment and how the app supports treatment and adherence. Having easy access to the exercise recommendations from the physical therapist through their own smartphone made it easy for patients to not only exercise as often as recommended, but also maintain proper form during the exercises. The push messages sent by the app as a reminder to perform the exercises, the option to set the reminder at a preferred time, and the video instructions of the exercises all contributed to patients’ confidence when exercising at home.

In a previous study, participants had no experience with digital technologies to support exercise adherence but were asked about their expectations regarding new technologies [12]. The patients were not very enthusiastic about the idea of reminder messages on their smartphones and expected them to be too intrusive. It is possible that in practice, it is important for a patient to use a new technology as part of treatment for some time before deciding on its added value. The participants in this study mentioned using this strategy to determine the usefulness of the app for themselves. Therefore, physical therapists should support patients with the shift toward the use of mHealth apps during treatment to allow patients to experience the benefits these new developments bring.

The last theme, “performance,” describes which functions of the app are most important according to the patients and how the performance of the app could be improved in the future. The video and text instructions, the reminders, and the option to self-monitor adherence were considered to be the most important functions of the app. Suggestions for future improvements were mainly aimed at making it even easier to use the app at home.

The findings of this study are similar to the results from studies on other mHealth or eHealth apps [23,24]. For instance, Svendsen et al reviewed the qualitative literature on digital interventions for the self-management of LBP [23]. After analyzing the included studies, 4 major themes were found: information technology (IT) usability and accessibility, quality and amount of content, tailoring and personalization, and motivation and support. A different review found that health status, usability, convenience and accessibility, perceived utility, and motivation were the main themes describing the barriers to and facilitators of engagement with remote measurement technology for health management [24].

Although the terminology describing the themes differs between studies, the content of the themes is broadly similar. For instance, “reminders and notifications,” “accessible at all hours and locations,” “easily accessible with low effort,” and “high user friendliness” were found to be facilitators for IT usability and accessibility in the study by Svendsen et al, whereas the themes “usability” and “convenience and accessibility” from the study by Simblett et al have similar facilitators [23,24]. In this study, the use of reminders, easy integration in daily life, and the high usability of the app contributed to its acceptability, corresponding with the findings from the previous studies. The high agreement between previous studies and this study, despite the different types of apps used by patients with different health problems, suggests that these findings can most likely be generalized between apps and health problems. This study adds to the findings that patients view the interaction between patients and physical therapists as vital when using an app as part of treatment. This suggests that Physitrack is well suited to support treatment but not to replace a physical therapist.

### Limitations and Trustworthiness

To put these results into perspective, several issues must be discussed. First, none of the included participants scored the usability of Physitrack lower than 70 (ie, acceptable) on the SUS. A possible explanation is that the physical therapists treating potential participants for the study only used Physitrack with patients they expected to benefit from the app. Patients who might have found the app unusable or who would not be able to use the app effectively might not have been offered the app as part of treatment.

A second limitation of the study was that the participants were relatively young, with just one exception. Older patients might not be able to use an app as effectively as younger participants. Similar to the first limitation, the physical therapists might not have offered the app to patients they expected would have no or little benefit from it. In addition to age, a patient might not have been suitable for treatment using an app for other reasons. Using an instrument, such as the “Dutch Blended Physiotherapy Checklist,” can assist physical therapists with deciding when to and when not to use an app such as Physitrack [25].

The last limitation is that the generalizability of the results in this study might be limited because of the specific app used and the inclusion of only patients with LBP in the study. However, the advantages of Physitrack mentioned by the patients relate mainly to features of the app and the patient-therapist interaction. Patients did not mention the cause of their complaints as having an impact on their acceptance of the app or how they used the app. Combined with the previously mentioned findings that barriers and facilitators related to the acceptance of mHealth apps do not seem to be impacted by a specific diagnosis, the results of this study can most likely be safely generalized to patients with other musculoskeletal disorders [20,23,24].

To increase the trustworthiness of data collection, prior to interviewing participants, the interviewer practiced the interviews and use of the interview guide with volunteers not participating in the study. The feedback from the volunteers helped to improve the thoroughness and consistency of the interviews. During data collection, a member check was performed by providing participants with a written summary of the interview and the opportunity to request changes or additions to their interviews to ensure its completeness. Furthermore, the use of the “Framework Method” methodology provided a transparent and rigorous method for data analysis [18].

### Implications

Physitrack appears to be a useful tool to complement physical therapists’ face-to-face treatment of patients with LBP. Although other mHealth solutions have displayed beneficial effects for patients with LBP and other musculoskeletal complaints, further research is required to investigate whether adherence to HBE interventions improves when using these apps during treatment [26-28]. Knowledge of the added value from Physitrack and similar apps to support HBE and the results of this study can support the implementation of these apps in clinical practice. The apparent importance of the physical therapist–patient interaction found in this study should be investigated further. Additional information on physical therapists’ perspectives regarding working with mHealth apps to support HBE and the effects of the physical therapist–patient relation on treatment results might lead to more effective treatments in the future. Although explorative research regarding the usability and acceptability of an app to support HBE by physical therapists is available, research involving physical therapists, patients, and their interactions when using smartphone apps to support HBE is still lacking and should be further investigated [29].

### Conclusion

Patients who used Physitrack accepted the app as part of treatment when it was easy for them to use, when it benefited their needs, and when the therapist instructed them in its use. Satisfaction is determined by the perceived support from the app when exercising at home and the perceived increase in adherence. Patients considered the video and text instructions, reminder functions, and self-monitor functions to be the most important aspects for the performance of the app during treatment. Physical therapists using Physitrack and similar apps to support HBE can use the findings from this study to effectively support their patients when exercising at home during treatment.

## Abbreviations

HBE: home-based exercise
IT: information technology
LBP: low back pain
mHealth: mobile health
SUS: System Usability Scale
